# Tranexamic acid combined with compression dressing reduces blood loss in gluteal muscle contracture surgery

**DOI:** 10.1186/s12893-022-01497-z

**Published:** 2022-02-11

**Authors:** Jun Ma, ZeYu Huang, Qiang Huang, ZongKe Zhou, FuXing Pei, Bin Shen

**Affiliations:** Department of Orthopedics Surgery, Orthopedic Research Institute, West China Hospital, West China Medical School, Sichuan University, 37# Guoxue Road, Chengdu, 610041 Sichuan Province People’s Republic of China

**Keywords:** Tranexamic acid, Blood loss, Complications, Gluteal muscle contracture

## Abstract

**Background:**

Blood loss and incision-related complications caused by the surgical procedure to release gluteal muscle contracture (GMC) put negative effects on the surgical outcomes. Current procedures to prevent blood loss and complications are not satisfactory. The current study aimed to determine whether tranexamic acid (TXA) in combination with pressure dressing reduce the amount of blood loss, the rate of incision-related complications, and the rate of readmission for patients undergoing surgeries to release GMC.

**Methods:**

49 GMC patients were finally included in the study and were randomly divided into two groups: study group and control group. Patients in both groups received minimally invasive surgery to release GMC except that in the study group, patients were administered a dosage of 20 mg/kg of intravenous TXA preoperatively, and 2 subsequent dosages of TXA at 10 mg/kg at two time points: 3 and 6 h after the first dose. Gauze soaked with TXA was used to pack the wound for 10 min before the incision closure. Then the wound was pressure-wrapped with a hip-spica bandage for 24 h after the surgery in the study group.

**Results:**

The level of UBL in the study group was significantly lower compared to that in the control group. Similar results were also found for UMHD and UMAD. The incision-related postoperative complications were greatly decreased in the study group compared to those of the control group as well. So was the 30-day readmission rate. All patients in both groups reached “excellent” or “good” level with respect to the postoperative function evaluation.

**Conclusions:**

Intravenous and topical application of TXA combined with 24 h pressure hip-spica bandage reduces perioperative blood loss, rate of incision-related complications, and the rate of readmission for GMC patients undergoing minimally invasive surgical releasing procedure.

*Trial Registration* Chinese Clinical and Trial Registry ChiCTR2000039216, registration date 2020/10/22, retrospectively registered

## Background

Gluteal muscle contracture (GMC) is a clinical syndrome characterized by the contracture of the gluteal muscles, such as gluteus maximus, medius and minimus, at various degrees. The main pathological features of GMC are degeneration, necrosis and fibrosis of gluteal muscles and fascia, which was firstly described by Fernandez de Valderrama in 1981 [1]. This disease was considered uncommon previously. But nowadays, it is found more prevalent than thought and exists throughout the world [1–11]. Despite the previous effort on pathogenesis and treatment methods of GMC, more studies are still needed to better understand the disease development and to more efficiently treat the disease to provide a better treatment outcome and a better prognosis. For instance, Zhou et al. recently found that miR-29a level was deregulated in the plasma and the contracture band of GMC patients, suggesting the role of miR-29a as a potential biomarker of GMC for early diagnosis and a potential targeting candidate to treat the disease [12]. Although non-surgical treatment strategies have been developed to treat GMC previously, the results of Zhao's study showed that the effect of non-surgical treatments alone was unsatisfactory even for those patients with a low level of contracture, not to mention for more serious cases [11]. Although new techniques have been developed to treat GMC such as arthroscopic release [10, 13], majority of surgical procedures to treat GMC are open release procedures at the current stage [14]. In general, surgical release is still the primary treatment for GMC with satisfactory outcomes [14].

Despite the advantages of surgery treatments for GMC, one common issue that needs to be addressed is the surgical site infection and incision-related complications, including but not limited to, hematoma, tissue swelling, delayed wound healing and wound exudation. Decreasing the incidence rate of such postoperative complications is greatly beneficial to improve the outcomes of the surgical procedures. It has been found that the incidence of these complications is associated with excessive blood loss during the procedure and/or poor ooze control after the procedure. Thus, preventing excessive blood loss could be an effective way to decrease the postoperative complication rate. According to the results of previous studies, reasons for perioperative blood loss include surgical trauma and hyperfibrinolysis [15, 16]. Thus, controlling the active bleeding and preventing hyperfibrinolysis during and after the surgical procedure are two key steps to decrease the blood loss. To achieve these goals, we decided to utilize tranexamic acid (TXA) in combination with compression dressing technique for the following reasons: (1) Compression dressing is a fundamental hemostasis technique that is widely used for reducing bleeding in various situations. It is relatively simple, easy to apply and cost effective. (2) Tranexamic acid (TXA) is an antifibrinolytic drug that has been proved functional in various type of surgeries at different degree regarding the prevention of excessive blood loss [15–21]. For example, intravenous TXA was effective on reducing blood loss and transfusion rate in periacetabular osteotomy [22]. The role of TXA in preventing excessive blood loss is extensively discussed by Ockerman et.al in her recently published review [23]. However, evidence for the role of TXA in GMC release surgeries is still lacking.

Based on the above-mentioned findings, we hypothesized that TXA in addition to compression dressing may reduce the blood loss caused by surgical procedures, and consequently, postoperative incision related complications in GMC patients. Therefore, we carried out the present study to investigate the efficiency of TXA plus compression dressing in perioperative bleeding reduction and its effect on incidence of complication in GMC patients. We found that compared to routine GMC release surgery, compression pressure on top of intravenous and topical TXA administration greatly reduced the blood loss, incision related complication rate, and readmission rate of patients after GMC surgery, suggesting that TXA in combination with compression dressing is an effective way to improve the outcome of GMC release surgery.

## Methods

### Study design

This study was a prospective, randomized clinical trial. Adult patients undergoing GMC release surgery in our center from January 2018 to December 2019 were enrolled in the current study. The research protocol was approved by the review board of our institution before the study started and registered in the Chinese Clinical and Trial Registry (ChiCTR2000039216). Written informed consent and research authorizations were obtained preoperatively from all participants.

### Patients

All patients scheduled to undergo GMC release surgery in our center were categorized by Zhao’s classification criteria [11]. Those criteria were summarized in Table 1. Patients were classified as GMC level 1 (GMC I), GMC level 2 (GMC II) and GMC level 3 (GMC III). All GMC level 2 (GMC II) patients were considered eligible for the current trial. Patients were randomly divided into two groups, the control group and the study group. Exclusion criteria for both groups included: (1) anemia (hemoglobin [Hb] level of < 120 g/L for female, < 130 g/L for male); (2) cardiovascular problems (history of myocardial infarction, angina, and atrial fibrillation); (3) clotting disorders, including any abnormality of platelets, activated partial thromboplastin time, prothrombin time and international normalized ratio; (4) allergy to TXA.

**Table 1:** Zhao’s criteria for GMC classification

Level 1 (GMC I)	GMC with mild manifestations, including less than 15° abduction contracture when hip and knee are both at 90° flexion, or less than 20° if hip or knee has no flexion. Weakly positive for Ober’s sign and frog squatting sign. No obvious limp gait shown when the lateral inclination of pelvis is less than 10° on the anterior–posterior radiograph
Level 2 (GMC II)	GMC with moderate manifestations, including 15° to 60° abduction contracture when hip and knee are at the 90° flexion position, or abduction less than 10° if hip and knee are at the extension position. Clearly positive for Ober’s sign and frog-leg squatting sign. Obvious limp gait is shown when the lateral inclination of pelvis on the anterior–posterior radiograph is from 10° to 20°
Level 3 (GMC III)	GMC with severe manifestations, including over 60° of abduction deformity when hip and knee joints are at 90° flexion, or no abduction if hip and knee joints are at the extension position. Strongly positive for Ober’s sign and frog squatting sign. Extremely obvious limp gait is shown when the lateral inclination of pelvis is over 20° on the anterior–posterior radiograph

All surgeries were performed by the same two surgeons using the same surgical technique. A third senior surgeon, who were blind to the surgical procedure, data collection and analysis performed patient administration process including surgical plan decision, medications, rehabilitation, discharge, and readmission.

### Surgical procedures and perioperative care protocol

A longitudinal skin incision centered on the greater trochanter (average 4 cm) was made after the patient was positioned in a lateral position. The soft tissue was kept isolated until a constriction zone was observed, which was mainly found in the tensor fascia latae and tractus iliotibialis, as well as the superior anterior part of the gluteus maximus. By taking the greater trochanter as the center, a “+” shaped incision was made in the constriction band which was fully released from the anterior–posterior direction and the longitudinal direction by the “moving windows” technique.

Ober’s sign was checked after the gluteus maximus and associated fascia were released. If it still existed, gluteus medium and gluteus minimus were further explored. 0.25% ropivacaine (40 ml) was administered by peri-articular injection. Incision would be directly closed once release was achieved. Hemostasis procedures were completed for patients in the control group by this step. For patients in the study group, a dosage of 20 mg/kg of IV-TXA was administered 5–10 min before skin incision. After release, the wound was packed with the gauze soaked with 1% TXA for 10 min before the incision closure, followed by pressure-wrapping with a hip spica bandage for 24 h after the surgery. Patients would also receive 2 doses of 10 mg/kg of IV-TXA 3 h and 6 h after the initial dose of IV-TXA.

No drainage was used in both groups. Once the patient returned to the inpatient ward, a cold pack was placed at the surgical site for 12 h. A discharge assessment was performed after removing the pressure dressing at 24 h postoperatively. The discharge criteria include normal body temperature and dry wound. The discharge of patients with wound complications would be postponed.

Daily function exercise started on the 1st morning postoperatively (POD1) in both groups. Function exercise started with hip flexion and extension, and model catwalk in the beginning, followed by sitting with leg crossing and squatting with leg being closed once the wound was dry and no hematoma found around the hip joint on day 2 postopeartively (POD2). All exercises after the pressure dressing removal were under the supervision and assistance of a physiotherapist. Follow-up was performed at the 1, 3 and 4 weeks after the surgery. Suture was removed 3 weeks after the surgery. Postoperative care protocol was carried out by nurses who were blind to patient’s group.

### Outcome measurements

Information about patient demographics, medical history, and concomitant medication, as well as the pre-operative routine examinations results, were collected. The pre-operative routine examinations included a whole blood test, liver function, renal function, coagulation function, and markers of inflammation (C-reactive protein [CRP] and interleukin 6 [IL-6]).

Whole blood tests, liver, and renal function tests were also performed at POD1, then tested every other day. The 30-day readmission for the wound complications, such as local hematoma, soft tissue swelling, blood exudation, fat liquefaction, delayed incision healing, and surgical site infection (SSI), was recorded. We compared total blood loss (TBL), maximum Hb drop and transfusion rate between the two groups. TBL was calculated by the Gross formula [24].

The postoperative function of all patients was ranked as “excellent,” “good,” “fair,” or “poor” based on Zhao’s and Guo’s criteria [5, 11] (Table 2). Patients was ranked based on gait, pelvic tilt, hip strength, range of motion, existence of GMC signs, and leg length discrepancy (LLD).

**Table 2:** Criteria for postoperative function assessment

Excellent	No snapping signs (neither subjective symptom nor objective sensation)**,** normal gait, no LLD or pelvic tilt, full hip range of motion and strength
Good	No snapping signs (neither subjective symptom nor objective sensation), normal gait, LLD < 1.5 cm, no pelvic tilt, nearly full functional hip (can squat down with closing legs, may cannot or difficultly sit with crossing legs)
Fair	No objective GMC signs, may slight subjective feeling of tension. Nearly normal gait, LLD < 2 cm, slight pelvic tilt, possible decreased hip range of motion or strength (cannot or difficultly squat down with closing legs and sit with crossing legs)
Poor	Presence of GMC symptoms or signs, LLD ≥ 2 cm, pelvic tilt, abnormal functional recovery of hip joint (range of motion and strength)

### Statistical analysis

Due to the lack of enough previous study results and the data from the GMC surgery in our own center, the total perioperative blood loss of an open unilateral GMC release surgery was about 280 ml. We estimated that TXA combined with pressure dressing could reduce bleeding by 80–100 ml. With the desired power of 0.80 and a significant level of 0.05, a sample size calculation requiring to compare two means (2-sample, 2-side equality) was performed by the demonstration from http://powerandsamplesize.com/Calculators/. It was estimated that a sample size of 32–50 patients in total (difference in reduced blood loss due to TXA between 80 and 100 ml) was needed.

All data was analyzed by using SPSS 26.0 software (SPSS Inc., Chicago, IL, USA). Independent t-tests were used for analyzing continuous variables including maximum decline, BMI, age, and other factors. Pearson Chi-square test or Fisher exact test were used to analyze categorical variables. *p* < 0.05 was considered statistically significant.

## Results

### Patients’ demographics

A total number of 62 patients were scheduled to undergo GMC release surgery in our institute during the patient recruitment time frame from January 2018 to December 2019. Among them, 49 level II patients were eligible for our study. These 49 patients were randomly divided into two groups, with 25 patients in the control group (6 unilateral, 19 bilateral) and 24 patients in the study group (2 unilateral, 22 bilateral). The baseline characteristics of the two groups were listed in Table 3 and were comparable.

**Table 3:** Baseline characteristics and intraoperative demographics

Demographic	Study group (n = 24)	Control group (n = 25)	*p* value
Age (y)	26.25 ± 6.68	25.68 ± 5.61	0.747
Gender (M/F)	9/15	10/15	0.861
Height (m)	1.62 ± 0.09	1.63 ± 0.08	0.496
Weight (kg)	54.98 ± 10.73	56.48 ± 11.35	0.637
BMI (kg/m^2^)	20.92 ± 2.50	20.99 ± 2.62	0.918
Sides	46	44	0.144
Bilateral	22	19	–
Unilateral	2	6	–
Pre-Hb (g/L)	142.63 ± 11.73	145.04 ± 16.22	0.555
Pre-HCT (L/L)	0.41 ± 0.03	0.44 ± 0.05	0.06
Pre-ALB (g/L)	48.18 ± 2.24	49.15 ± 2.87	0.193
PBV	3678.59 ± 748.73	3783.86 ± 757.08	0.627

*BMI* body mass index, *PBV* patient blood volume, *Pre* preoperative, *Hb* hemoglobin, *HCT* hematocrit, *ALB* albumin

### Blood loss

The postoperative parameters were summarized in Table 4. As seen in Table 4, the average total blood loss (TBL), TBL rate, maximum Hb decline (MHD), and MHD rate in the study group were lower than those in the control group but the difference was not statistically significant. Considering the difference between bilateral and unilateral surgery on blood loss, and the fact that some patients underwent unilateral GMC release surgery, while others had surgery performed bilaterally in the present study, all data of blood loss were converted to one side for the whole group comparison. After data conversion, the results showed that patients in the study group had an average of unilateral blood loss (UBL) of 212.40 ± 84.22 ml, which was significantly lower than the average blood loss of 285.26 ± 126.62 ml in the control group (*p* < 0.05). Similar results were found in terms of unilateral maximum Hemoglobin decline (UMHD), and unilateral maximum albumin decline (UMAD) which were 7.52 ± 2.84 g/L and 3.55 ± 1.29 g/L in the study group, and 9.62 ± 3.75 g/L and 5.34 ± 2.33 g/L, respectively (*p* < 0.05) (Table 4).

**Table 4:** Postoperative parameters

Variable	Study group	Control group	*p* value
TBL (ml)	398.92 ± 158.37	505.23 ± 273.57	0.104
TBL rate (%)	11.08 ± 4.04	13.62 ± 7.42	0.145
*UBL (ml)	212.40 ± 84.22	285.26 ± 126.62	0.022^#^
*UBL rate (%)	5.89 ± 2.15	7.69 ± 3.35	0.031^#^
MHD (g/L)	14.13 ± 5.32	16.92 ± 8.29	0.169
MHD rate (%)	9.86 ± 3.30	11.64 ± .54	0.179
*UMHD (g/L)	7.52 ± 2.84	9.62 ± 3.75	0.033^#^
*UMHD rate (%)	5.25 ± 1.81	6.64 ± 2.49	0.032^#^
MAD (g/L)	6.65 ± 2.17	8.71 ± 2.69	0.005^#^
MAD rate (%)	13.81 ± 4.50	17.61 ± 4.87	0.005^#^
*UMAD (g/L)	3.55 ± 1.29	5.34 ± 2.33	0.002^#^
*UMAD rate (%)	7.36 ± 2.65	10.77 ± 4.44	0.002^#^
PLOH (d)	2.46 ± 0.93	2.64 ± 0.81	0.469

*TBL* total blood loss, *UBL* unilateral blood loss, *MHD* maximum Hb decline, *UMHD* unilateral maximum Hb decline, *MAD* maximum ALB decline, *UMAD* unilateral maximum ALB decline, *PLOH* postoperative length of hospital stays

*The data on blood loss are averaged to single side for comparison, # *p* < 0.05 was considered statistically significant

### Postoperative function

According to the criteria listed in Table 2, the postoperative function of all patients was ranked “excellent” or “good” (Table 5). All patients’ gait returned to normal, and the snapping signs disappeared. Five patients (2 in the study group and 3 in the control group) were found difficult to sit with crossing legs and even unable to do it with assistance, while other patients could easily complete it. Another four patients were found with the leg-length discrepancy, which was not obvious when they were in standing position or when they walked. The effectiveness of treatment in terms of function restoration was 100% given that the effectiveness was defined as the postoperative function reached “excellent” or “good”. The excellent, good rates were 91.67%, 8.34% in the study group, and 88%, 12% in the control group, respectively. There were no statistical differences between the two groups (*p* = 0.672).

**Table 5:** Postoperative Function

Functional grade	Study group (Effective rate)	Control group (Effective rate)	p value
Excellent	22 (91.67%)	22 (88%)	0.672
Good	2 (8.34%)	3 (12%)	
Fair	0	0	
Poor	0	0	

### Complications

A total number of 12 patients, experienced incision-related complications including 2 in the study group and 10 in the control group (Table 6). One patient in the study group had fat liquefaction, and another patient had soft tissue swelling and redness around the incision in the first week after surgery. The complications disappeared in these 2 patients soon after dressing change. A similar scenario occurred in 6 patients in the control group. However, due to persistent wound exudation, incision healing was delayed in 4 out of the 6 patients in the control group. They had to be readmitted for re-suturing the incision in 2–3 weeks after surgery. Readmissions occurred within 5–7 days after surgery.

**Table 6:** Postoperative incision-related complications

Group	Complications		Readmission
Study	Fat liquefaction		–
Study	Soft tissue swelling		–
Control	Soft tissue swelling	Fat liquefaction	–
Control	Blood exudation	Incision delayed healing	Re-suture
Control	Soft tissue swelling		–
Control	Soft tissue swellings		–
Control	Hematoma and exudation	Incision delayed healing	Re-suture
Control	Soft tissue swelling		–
Control	Soft tissue swelling		–
Control	Blood exudation	Soft tissue swelling	–
Control	Incision delayed healing	Blood exudation	Re-suture
Control	Incision delayed healing	Blood exudation	Re-suture

If the soft tissue swelling was excluded from the list of postoperative complications (only objective conditions such as liquefaction and exudation were counted), the incidence of complications in the study group was 4.17% (1/24) compared to the 24% (6/25) in the control group. No patient had nerve injury, blood vessel damage, or abductor weakness. There were no statistical differences in PLOH between the two groups (*p* = 0.469).

## Discussion

In the present study, we investigated the effect of TXA in combination with compression dressing on preventing excessive blood loss and consequent incision related complications in GMC release surgeries. The results demonstrated that TXA combined with compression dressing greatly reduced the amount of blood loss and incision related complications after surgical release of GMC, suggesting the potential of the combination treatment to improve the GMC surgery outcomes, as well as other applicable surgeries.

As the primary treatment for GMC, surgery inevitably cause bleeding and surgical complications compared to non-surgical, conservative treatments. How to reduce blood loss and the incidence of complications are two issues that clinicians are facing. Although few studies focused on the effect of TXA in GMC releasing surgeries, the role of TXA in reducing bleeding has been proved in other orthopedic surgeries [25–29]. However, the regimen used to administer TXA varied in different studies. Based on the facts that administration of TXA via different route, intravenously or topically, reduced the blood loss perioperatively, we tried applying TXA both intravenously and topically.

Furthermore, perioperative blood loss includes intraoperative bleeding and hidden blood loss (HBL). With the decrease of HBL in the joint, the chances to reduce joint swelling would be increased [30]. Studies performed in total knee arthroplasty (TKA) have demonstrated that the HBL could be as much as several hundred milliliters [15, 16, 31], which could be up to half of the total blood loss for the whole procedure. Similarly, we found that HBL could not be underestimated in GMC surgery as well. Based on our clinical practice experience, HBL in GMC could cause hematoma, wound exudate, aggravate postoperative soft tissue swelling and pain, which will all eventually have an impact on the complication incidence. Both our previous study [15, 16] and the study conducted by Blanié et al. [32] showed that the postoperative fibrinolysis would last 18–24 h. Therefore, we designed the subsequent administration of two doses of 10 mg/kg TXA 3 and 6 h after the 1st dose of TXA in the study group. The results demonstrated that administration of TXA both pre and post operatively via different route significantly reduced blood loss without obvious side effects, suggesting the potential combinational use of TXA via different route and at different time points.

In addition to TXA, a difference between our study and other studies was the usage of compression dressing, which was supposed to add an additional physical control of blood loss. The results were satisfactory, indicating the role of compression dressing in bleeding control postoperatively. More well-designed studies are needed to investigate the efficiency of compression dressing on reducing bleeding by treating patients with or without compression dressing.

Few previous studies on GMC surgery focused on total perioperative blood loss [1–11]. The reason might be that GMC releases surgeries, whether open surgery or arthroscopy, causes less trauma, less blood loss, and lower chance of blood transfusion requirements compared to other orthopedic operations, such as fracture fixing or joint replacement. However, the results in the current study clearly demonstrated that a combination of TXA treatment and pressure dressing in GMC release surgery was extremely valuable.

Blood loss is an indicator for tissue trauma [33]. Less severity of the tissue trauma means less pain and better patient experience. Additionally, bleeding and exudation of the wound would affect postoperative plasma albumin level, which is important in regulating blood volume by maintaining the oncotic pressure of the blood compartment [34]. Tissue edema, which will exacerbate inflammatory responses caused by surgical trauma, is associated with reduced albumin level. In the present study, MAD, MAD rate, UMAD, and UMAD rate in the study group were statistically lower in the study group compared to those in the control group, suggesting less albumin loss in the study group. Consistently, less complications were found in the study group, which was in line with previous findings. In the present study, the incidence of complications (24%) was significantly higher in control group compared to that in study group (4.17%). If using readmission and reoperation rate as indexes, the difference was even more significant. As the result shown, the 30-day readmission and the reoperation rate were 16% (4/25) in the control group, and no above-mentioned situation existed in the study group. Obviously, TXA combined with compression dressing played an important role in reducing bleeding, thus reducing local complications related to incision.

The present study demonstrated several advantages. First, as a randomized controlled trial that was carefully designed and strictly executed, the deviation of the data was small. Second, all patients were administered by the same senior surgeon who decided the surgical plan, guided the perioperative medications, designed the rehabilitation exercise program, and evaluated the patient’s discharge and readmission criteria, making the data in both groups less variated. Third, the surgeon was blind to surgical procedures, data collection and analysis, which reduced bias as much as possible.

Limitations of the current study are as follows: first, although the difference between the two groups was intuitively obvious from the numerical values regarding the complication rate, it needs to be cautious to draw conclusions on other investigated parameters since the sample size used in the present study was calculated for the blood loss evaluation. One needs to pay more attention to the sample size to evaluate the results carefully and thoroughly with respect to other parameters. Second, the follow-up period only lasted 4 weeks after the surgery. Due to the relatively short time of follow-up, we were unable to investigate the recurrence rate of GMC after the surgery. A future investigation with a bigger sample size, longer follow-up duration and separated TXA and compression dressing treatment is needed to further illustrate the efficacy of TXA on reducing bleeding in GMC surgeries.

## Conclusion

Intravenous and topical application of TXA combined with 24 h pressure hip-spica-bandage could significantly reduce perioperative blood loss in invasive surgical release of GMC as well as the incision-related complications and patient readmission rate.

## Data Availability

The datasets used and/or analyzed during the current study are available from the corresponding author on reasonable request.

## Abbreviations

GMC: Gluteal muscle contracture
TXA: Tranexamic acid
TBL: Total blood loss
UBL: Unilateral blood loss
MHD: Maximum Hemoglobin decline
UMHD: Unilateral maximum Hemoglobin decline
MAD: Maximum albumin decline
UMAD: Unilateral maximum albumin decline
PLOH: Postoperative length of hospital stay
